# Gelotophobia and the Challenges of Implementing Laughter into Virtual Agents Interactions

**DOI:** 10.3389/fnhum.2014.00928

**Published:** 2014-11-18

**Authors:** Willibald F. Ruch, Tracey Platt, Jennifer Hofmann, Radosław Niewiadomski, Jérôme Urbain, Maurizio Mancini, Stéphane Dupont

**Affiliations:** ^1^Personality and Assessment, Department of Psychology, University of Zurich, Zurich, Switzerland; ^2^InfoMus Laboratory, Dipartimento di Informatica, Bioingegneria, Robotica e Ingegneria dei Sistemi, University of Genova, Genova, Italy; ^3^TCTS Laboratory, Numediart and InforTech Research Institutes, Faculté Polytechnique de Mons, University of Mons, Mons, Belgium

**Keywords:** gelotophobia, laughter, social phobia, virtual agent

## Abstract

This study investigated which features of AVATAR laughter are perceived threatening for individuals with a fear of being laughed at (gelotophobia), and individuals with no gelotophobia. Laughter samples were systematically varied (e.g., intensity, laughter pitch, and energy for the voice, intensity of facial actions of the face) in three modalities: animated facial expressions, synthesized auditory laughter vocalizations, and motion capture generated puppets displaying laughter body movements. In the online study 123 adults completed, the GELOPH <15 > (Ruch and Proyer, 2008a,b) and rated randomly presented videos of the three modalities for how malicious, how friendly, how real the laughter was (0 not at all to 8 extremely). Additionally, an open question asked which markers led to the perception of friendliness/maliciousness. The current study identified features in all modalities of laughter stimuli that were perceived as malicious in general, and some that were gelotophobia specific. For facial expressions of AVATARS, medium intensity laughs triggered highest maliciousness in the gelotophobes. In the auditory stimuli, the fundamental frequency modulations and the variation in intensity were indicative of maliciousness. In the body, backwards and forward movements and rocking vs. jerking movements distinguished the most malicious from the least malicious laugh. From the open answers, the shape and appearance of the lips curling induced feelings that the expression was malicious for non-gelotophobes and that the movement round the eyes, elicited the face to appear as friendly. This was opposite for gelotophobes. Gelotophobia savvy AVATARS should be of high intensity, containing lip and eye movements and be fast, non-repetitive voiced vocalization, variable and of short duration. It should not contain any features that indicate a down-regulation in the voice or body, or indicate voluntary/cognitive modulation.

## Introduction

Virtual environments and virtual agents become more and more popular in various domains, such as e-learning [e.g., Zaíane (2002)], intervention programs [e.g., Rinck et al. (2010)], games [e.g., Adobbati et al. (2001)], and websites [i.e., in online shops; e.g., Grenci and Todd (2002)]. Thus, various fields are concerned with how such environments and the virtual characters that interact within them can be made more natural and rewarding. As one attempt of trying to increase the pleasurable component of interacting with a virtual agent, increase the engagement, and enhance the communication outcome, smiling and laughter as expressive features of positive affect have been implemented [e.g., Niewiadomski et al. (2010), Ochs et al. (2012), Hofmann (2014), Hofmann et al. (under review)]. The functions of smiling and laughter are highly significant for social interactions and communication [e.g., Chapman (1983), Glenn (2003), Holt (2010)]. Thus, any virtual interface, performing the role of a companion, tutor, or simply programed to interact with human beings, will considerably benefit from being able to utilize smiling and laughter correctly, as well as to detect such displays and respond to them adequately.

While for most individuals the interaction with a laughing virtual agent will be conducive to positive affect (through mimicry and emotional contagion), for some individual’s negative effects are to be expected. Individuals with a fear of being laughed at [gelotophobia, a universal phenomenon related to, but sufficiently distinct from social phobia; see Ruch and Proyer (2008a,b)] do not appreciate laughter, but see it as threatening and as a weapon [for a review, see Ruch et al. (2014)]. Thus, it is assumed that the same would hold true for gelotophobes when confronted with the laughter of a virtual agent. As such, the attempts to make virtual encounters more natural and rewarding by implementing smiles and laughter may lead to an aversive experience and a breakdown of the interaction for gelotophobes. Therefore, the responses of gelotophobes to virtual laughter should be studied to (a) find out how virtual laughter can be programed to be “gelotophobia-friendly,” (b) specify the fear triggers in virtual agent laughter for a better insight in the phenomenon of gelotophobia, (c) eventually develop programs that help gelotophobes to train a re-evaluation of laughter by training them with laughing virtual agents. Indeed, using virtual agents to investigate social cognitive disorders has been proven useful, for example, a study on social anxiousness (Vrijsen et al., 2010) showed that in general, individuals high in social anxiety did not appreciate the subtle mimicry behavior of virtual agents. Conversely, having a programmable AVATAR with precise known triggers levels that can be decreased over time as a desensitization tool, may be useful for cognitive behavioral therapies for the treatment of gelotophobia. Therefore, the current study investigates the fear triggers in virtual agents across different modalities (face, voice, and body) for gelotophobes and individuals without a fear of being laughed at.

As yet, the current DSM (DSM-IV TR, 2000) does not recognize gelotophobia as a diagnosable condition. It was primarily observed in interactions between therapist and patient. An article by Titze (2009) described vignettes of interactions with clients in a clinical setting who expressed concerns relating to fearing laughter and who held the belief that they were indeed credible objects of derision. These descriptive criteria allowed Ruch and Proyer (2008a,b) to build an efficient 15-item self-report instrument (GELOPH <15 >) that allowed for identifying gelotophobes at four different levels with none, slight, pronounced, and extreme fear of being laughed at. This shows gelotophobia is best conceptualized on a continuum where at the higher levels of this continuum gelotophobes consistently anticipate the shame induced by all laughter, even friendly laughter, in a fearful and negative way, making the assumption that it is malicious. Cross-cultural investigations show gelotophobia to be a universal phenomenon (Ruch et al., 2014).

For example, Forabosco et al. (2009) investigated gelotophobia. The authors went through the literature including the terms *humor/laughter* and *psychiatric patients* and identified very few studies dealing with both phenomena. The authors thus concluded, “the capability to positively experience humor and laughter are often compromised in psychiatric disorders, though in a somewhat different way” (p. 236). They then tested patients with (1) personality disorders; (2) schizophrenic disorders; (3) mood disorders; (4) anxiety disorders; (5) eating disorders with the Italian version of the GELOPH <15 > to investigate if gelotophobia was more prominent in psychiatric patients. The overall prevalence of gelotophobia was found to be higher among the psychiatric patients and highest for personality disorders and schizophrenic disorders. This heightened level of gelotophobia among psychiatric patients was also found in a Russian sample (Ivanova et al., 2012). Furthermore, the patients who had been in psychiatric care for longer than 5 years were found to have significantly higher levels of gelotophobia. Broadening the scope of psychiatric disorders, Weiss et al. (2012) found a partial overlap between Cluster A personality disorders and also schizoid and schizotypal personality disorders.

Carretero-Dios et al. (2010) investigated the fear of being laughed at and social anxiety. They showed that there were definite overlaps, yet there were still unique qualities to gelotophobia, which could not be accounted for by the measures of social phobia. Similarly, Edwards et al. (2010) found that gelotophobia was strongly related to, but still distinct from, social phobia. By taking a closer look at a sample of extreme gelotophobes, three distinct components within gelotophobia were separated. Platt et al. (2012) were able to clearly demonstrate what the distinctions were, as well as what the similarities were, compare to social phobia. The first factor was “coping with derision.” Gelotophobes cope by either controlling their environment and situations they are in, to ensure no one is laughing at them, or by actually internalizing the belief that they are a valid object of derision, thus, reconciling that they will be laughed at, or by social withdrawal. This latter facet is the one which links to known behaviors associated to social phobia (Rapee and Spence, 2004). However, gelotophobes also have two further components, these being a “paranoid sensitivity to anticipated ridicule” with paranoid sensitivity here referring to gelotophobes’ suspicion and belief that they will be the targets of laughter, even when this is unsubstantiated. The third factor is “disproportionately negative responses toward being laughed at,” which is also specific to gelotophobia, just as the factor “coping with derision.”

Therefore, it is legitimate to investigate this construct without the need to further investigate other aspects of social phobia, especially with the advent of computerized systems where laughter will be integrated into the interface. Still, we included a measure of social anxiety in the current study to see whether the effects of gelotophobia would still remain after controlling for social anxiety.

Gelotophobes have already been shown to perceive laughter differently to individuals with no fear of being laughed at (Ruch et al., 2014, 2015). Also, they have been shown to display fewer facial expressions of joy (Duchenne smiles and laughs) in an interview on positive emotions compared to the non-gelotophobes (Platt et al., 2013). This effect was found to be stronger for both the frequency and the intensity of joy smile responses toward laughter-eliciting enjoyable emotions than for the non-laughter-eliciting enjoyable emotions. Those who do not have gelotophobia responded positively, displaying smiles more strongly to laughter-eliciting than to no laughter-eliciting enjoyable emotion expressions. However, individuals with a pronounced level of gelotophobia showed the reverse pattern, displaying less joy smiles to the laughter-eliciting emotions. Therefore, those with gelotophobia may indeed jeopardize situations where the elicitation of positive affect or laughter is required. Furthermore, gelotophobes lack the ability to clearly judge the positive role of laughter and overly judge facially expressed joy to containing contempt, compared to individuals with no fear (Hofmann et al., under review). Yet, nothing is known about which features of the expression of laughter are perceived as malicious, as laughter is far more than simply a facial expression and also includes a host of body movements and vocalizations (Ruch and Ekman, 2001).

For the purpose of the current study, we investigated laughter expressed through face, voice, and body to identify which laughter features are perceived as malicious and consequently identify features that should be avoided when creating gelotophobia-friendly laughter. We manipulated synthesized laughter along theoretically derived dimensions and showed them to a sample of individuals with and without gelotophobia. The participants should rate the laughter on the dimensions of maliciousness, friendliness, and realness, as well as describe which features make a given laugh friendly or malicious in an open answer format. In the next paragraphs, we describe our hypotheses on each laughter modality separately.

The most extensively studied aspects of gelotophobes’ responses to laughter have been with facial expressions (e.g., Ruch et al., 2014; Hofmann et al., under review). Yet, facial displays of joy where always investigated holistically, and only one study targeted single facial features [and their intensity; see Hofmann (2014)]. In the current approach, joyful laughter stimuli in different intensities are utilized. For the gelotophobes, we expect that low and medium intensity laughs are perceived as more malicious compared to high-intensity laughs, as the former may give the impression of being cognitively regulated. This is because of the expressions occurring in different areas of the brain (Rinn, 1984). Emotion expressions, namely, the observable verbal and non-verbal behaviors that communicate an internal emotional or affective state, are quicker to appear than those representing cognitively motivated displays. Therefore, one can assume that the slower the modality the more it will appear as cognitive. In this instance, cognitive laughter expression refers to an expression that is voluntarily produced. For the non-gelotophobes, we expect that the ratings are primarily a function of the laughter intensity (stemming from perceptual studies and FACS codes). Thus, we expect that the effects of the stimulus intensity (low, medium, and high) on the ratings of maliciousness, friendliness, and realness will be different for the gelotophobia and non-gelotophobia groups.

In terms of body movements, no psychological study has investigated the perception of laughter body movements in gelotophobia yet. Wallbott (1998) investigated the effect of movement intensity on perceived valence/intensity and found that the ratings were predicted by the movement intensity. Thus, we choose intensity as an initial differentiating dimension to judge body movements of laughter. Five stimuli of low intensity and five of high intensity are rated. We assume that gelotophobes will generally perceive body movements of laughter as more malicious and less friendly than non-gelotophobes. Concerning the qualitative analysis of the laughter body movement features, we expect that movements that seem cognitively modulated, regulated, or restrained, lead to higher perceived maliciousness.

For the auditory laughter displays, we generated hypotheses based on the results of a pilot study (gelotophobes were asked to identify maliciousness indicators of laughter and they nominated features like laughter energy and pitch) and knowledge on vocal laughter perception in general. We hypothesize that in general the modifications that give the impression that the synthesized laughter sound is cognitively regulated will be the ones, which lead to higher maliciousness ratings, compared to the original laugh. In line with past research, we expect that the variability in pitch and rhythm influence the maliciousness, friendliness, and realness ratings [see also Bryant and Aktipis (2014) and Kipper and Todt (2001, 2003)]. We expect that compared to the original, sounds with modulated fundamental frequency (F0) variability, “slower” sounds and sounds with decreased intensity should be perceived as more malicious, less friendly, and less real compared to the original [see also Bryant and Aktipis (2014)]. Furthermore, we expect a general trend of gelotophobes rating auditory laughter stimuli as more malicious and less friendly [see also Ruch et al. (2009)].

## Materials and Methods

### Participants

The sample consisted of 123 participants partially completed the survey (at least one modality) and 71 participants completed all three modalities. All participants were English-speaking adults. The overall gender distribution was 78 males and 122 females. Ages ranged from 18 to 73 years (*M* = 33.80; SD = 13.37). Ten percent of participants had a secondary school education, 21% had a gymnasium/high-school education. The majority (37%) had a university degree and 23% reached a post-graduate degree. Those with an apprenticeship or had been educated in a technical college accounted for 3% of the sample. Participation of the survey was through their personal Internet access.

### Instruments

The *GELOPH* <15 > (Ruch and Proyer, 2008a,b) is a questionnaire assessing the level of the fear of being laughed at (i.e., gelotophobia) consisting of 15 items in a 4-point answer format (1 = *strongly disagree* to 4 = *strongly agree*). A sample item is “When others laugh in my presence I get suspicious.” Cronbach’s alpha (0.89) in the present sample is comparable to the English norm sample (α = 0.90; Platt et al., 2009).

The *social phobia inventory* (SPIN; Connor et al., 2000) is a 17-item self-report questionnaire, which assesses symptoms of social phobia on 3 dimensions (fear, avoidance, and physiological arousal). Respondents are required to answer how much they were bothered by particular symptoms during the past week, measured on a 5-point scale ranging from 0 (*not at all*) to 4 (*extremely*). The SPIN has good internal consistency and discriminant validity.

#### Procedure

##### Material production

Stimuli were produced for each modality separately (face, voice, and body). The basis for all animations was joyful laughs elicited by amusement [corpora by Urbain et al. (2010) and Niewiadomski et al. (2013)]. The specific criteria for each modality are reported in the respective section.

##### Face

In order to generate the virtual agents’ facial expressions, several steps were required. The first step was to choose six episodes of joyful laughter (two low, two medium, and two high intensity) of the freely available AVLC corpus (Urbain et al., 2010) by three authors. The selection criteria were (a) equal distribution of intensity levels [as rated by naive participants on the laughter globally, see Niewiadomski et al. (2012a,b) and assessed by the intensities of the AUs], (b) smooth animation, (c) good visibility of the face (i.e., no extreme head turns or head up/down). Each episode contains precisely one laugh. All laughs were voiced laughs of durations between 2 and 7 s. All the episodes were of one female subject (“Subject 5” in AVLC corpus).

**Figure 1:**
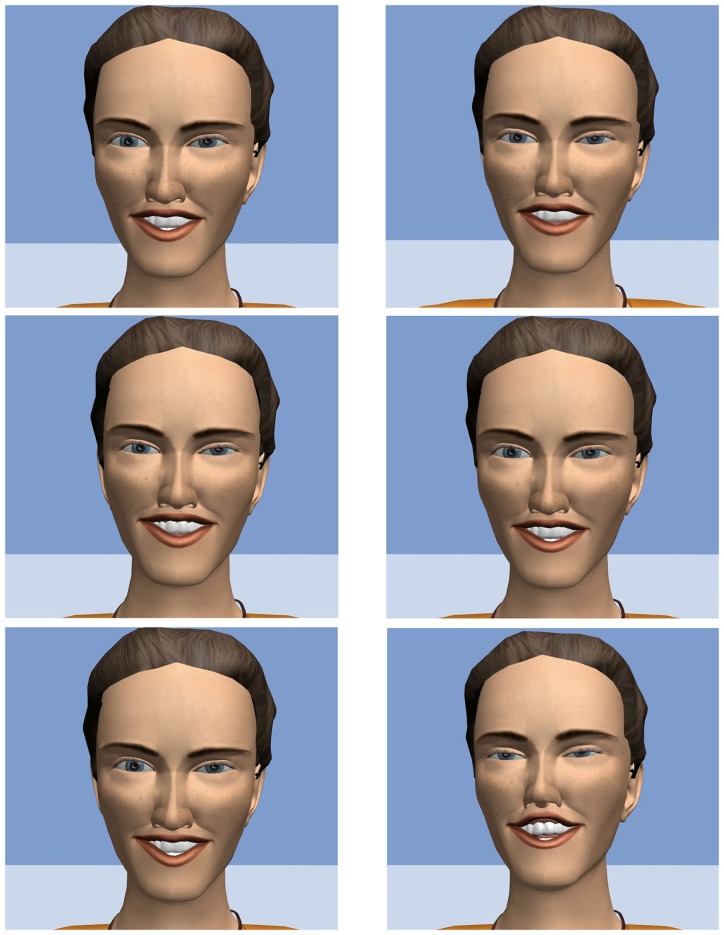
**Apex of the laughter events of the six stimuli**. Top row: two low-intensity laughs (left side: AU6B, AU12B, and AU25C; right side: AU6B, AU12B, and AU25B). Mid row: two medium intensity laughs (left side: AU6C, AU12C, and AU25C; right side: AU6C, AU12C, and AU25C). Bottom row: two high-intensity laughs (left side: AU4C, AU6D, AU12D, AU25D; AU6E, AU12E, and AU25E).

Next, the facial expressions of the chosen videos were processed with a freely available facial tracker (Saragih et al., 2011) and the tracked 2D data were retargeted onto the mesh of a virtual character [details of the procedure can be found in Qu et al. (2012)]. The animations resulting from the retargeting were additionally modified to enhance the visibility of the AU6 (cheek raiser), a marker of amusement. In particular, the intensity of AU6 in the final animation is proportional to the intensity of AU12 [details of the approach can be found in Niewiadomski and Pelachaud (under review)]. In the last step, the wrinkles associated to action units were applied to the virtual model according to the model proposed in Niewiadomski et al. (2012b) [see also Niewiadomski and Pelachaud (under review)]. The appearance and intensity of an expressive wrinkle depends on the intensity of the corresponding AU. Figure 1 shows the six facial laughter stimuli, depicting the apex of the laughter and its respective FACS codes.

Figure 1 shows the apexes of each of the facial expression stimuli separately.

##### Voice

The acoustic laughter synthesis process used for this study follows the approach described in Urbain et al. (2013). It relies on hidden Markov models (HMMs) to capture the statistical distributions of audio features (characterizing the shape of the sound wave) for each laughter phone (e.g., “h,” “a,” “e,” etc.). HMMs have the advantage to model the evolution of acoustic features both during each phone (thanks to the use of several states to model each phone) and across the laugh (thanks to the incorporation of derivatives in the feature set). Furthermore, the HTS toolbox (Oura, 2011) provides convenient ways to train contextual HMMs, meaning that different statistical distributions will be computed for each context the considered phone can be in, for example, the phone “h” will be associated to different distributions when it is followed by “a” or “e.” To synthesize a laugh, its phonetic transcription is provided to the trained HMMs. Although phonetic transcriptions are the only required parameter, HMM-based laughter synthesis also enables to easily control the duration of each phone as well as the fundamental frequency (pitch) pattern^1^.

For this study, laughs varying along four dimensions were synthesized: intensity, rhythm, fundamental frequency, and number of syllables. For each dimension, the starting point is a laugh synthesized from a human phonetic transcription and respecting the duration of each of the phones from the human laugh. Two female laughs (around 6.5 s each) and two male laughs (lasting around 2 and 5 s) were created that way, and were used for the four types of modifications presented below. They are called “base synthesis” laughs. All the laughs were voiced. Thus, 4 original laughs and 32 modified laughs were created^2^.

##### Body

Animations of laughter body movements were created using motion capture data of the multimodal multiperson corpus of laughter in interaction (MMLI corpus; Niewiadomski et al., 2013)^3^ and the Eyesweb XMI software platform (Eyesweb). For the purpose of this study, 10 episodes lasting between 11 and 30 s and involving 4 participants were chosen (5 annotated as low and 5 as high-intense laughter).

Data were collected using Xsens MVN Biomech System bodysuits (xsens), each of them consisting of 17 inertial sensors placed on Velcro straps. Data were captured at 120 frames per second; each frame consisting of 22 body joints’ location and rotation in a 3D reference space. An animated stick figure was created with the freely available Eyesweb XMI software platform (Eyesweb), starting from the 3D Xsens position data of the corresponding episodes. The advantage of such simple body visualization is that body movements are displayed precisely, avoiding that the lack of other modalities (e.g., facial expressions) would be perceived as awkward. In the stick figure animations, a frontal camera was used and the whole body is visible throughout the entire animation.

##### Study procedure

Data were collected online with the data collection platform “Unipark.” Participants were recruited over mailing lists of universities, forums, and social media platforms. After a welcome page that thanked people for their participation, instructions were given that advised the participant to allow cookies and to ensure having the brightness of the screen and the sound turned up. Further instruction was given to wear headphones for the duration of the study. All participants had to tick a confirmation box that they (a) had read the instructions, and (b) were wearing headphones, before being asked to continue with the study.

The participants were next given instructions to the nature of the study: “You will now be presented with an AVATAR. The laughter the AVATAR displays has been animated directly from a real situation where laughter occurred during a conversation between two people. The clip you are about to see shows a laugh, which occurred specifically during a conversation. This type of laughter happens when the person speaking is about to change the topic of conversation. This laugh is very common when people are talking either face-to-face or on the telephone.” Please focus on the facial expressions of the laughing AVATAR. The example of the instruction for participants in the hilarious laughter condition is “You will now be presented with an animated AVATAR. The laughter the AVATAR displays has been animated from a recording of a real situation where laughter occurred when the person laughing has found something to be very funny. The clip you are about to see shows a laugh, which occurred specifically during a conversation. For example, when the person was told a funny joke. This laughter is not so common and only happens in response to something that is hilariously funny.” All participants were then told to “Please focus on the facial expressions (auditory laughter/body movements) of the laughing AVATAR”^4^.

Next, the demographic questions, relating to gender, age, education, and which language was their mother tongue were asked. A further two questions were given, one relating to experiencing hearing problems or having previously taken part in a laughter perception study. Next, participants completed the GELOPH <15 > and the SPIN. All modalities (i.e., the 6 faces, 32 vocalizations, or 10 body movement) were presented in blocks (and randomized within blocks) but each participant was randomly assigned to being presented with either the face, voice, or body stimuli block first (with the other two modalities presented after completion of the first, and also randomized). Each video or audio clip was presented on one single page. A set of questions relating to how the clip was perceived was presented on the same page as each clip. These were (1) how malicious (with bad intention) is this laughter? (2) How friendly (with good intentions) is this laughter? (3) How real is this laughter? These were all rated on a 9 point Likert scale from 0 (not at all) to 8 (extremely). An open question was also asked (4) which markers in the face/voice/body lead to your perception of friendliness or maliciousness? Where the participant could type a response using 200 characters in a text box. The video or audio clip was automatically played as the participant clicked through each page. However, the video could be replayed as many time as required by the participants. After having rated the stimuli of all modalities, participants answered some control questions and were thanked for the participation. An email was offered for anyone requiring further information on the study or a post study brief report. This study conformed to the requirements for the approval of University of Zurich ethics committee.

## Results

### Analysis of gelotophobia

The averaged GELOPH <15 > total scores were computed. Participant’s gelotophobia scores ranged from 1.00 to 4.00. The distribution of scores for gelotophobia was *M* = 1.73, SD = 0.65. The cut off for gelotophobia was applied (i.e., 2.5) and yielded 71% (*n* = 87) with no fear, 22% (*n* = 27) were borderline, 6% (*n* = 8) were slight and 0.8% (1) indicated marked gelotophobia. Furthermore, gender was not related to gelotophobia, but age was negatively related *r*(123) = −0.241, *p* < 0.01 in the present sample.

### Analysis of the perception of facial laughter expressions

We computed three separate ANOVAs with the gelotophobia as a group factor (gelotophobia vs. no gelotophobia), the stimulus intensity as repeated measures (low, medium, and high) and maliciousness, friendliness, and realness as the dependent variables. Depending on the kind of expectations, main effects for intensity (for the two groups separated) and subsequent *post hoc* tests, or trend analyses (linear and quadratic trends) were computed.

The two stimuli of each intensity level were averaged. We investigated the perception of maliciousness first. As expected, among non-gelotophobes intensity of the facial display degree did not impact on level of perceived maliciousness, *F*(2, 140) = 0.15, *p* = 0.859 (*M*_low_ = 3.22, SD_low_ = 1.47, *M*_med_ = 3.32, SD_med_ = 1.76, *M*_high_ = 3.22, SD_high_ = 1.53). Gelotophobes, however, were sensitive to the intensity of the display, *F*(2, 58) = 4.77, *p* = 0.012, η_p_^2^ = 0.141. *Post hoc* tests revealed that the medium intensity (*M*_med_ = 4.03, SD_med_ = 1.61) was perceived as more malicious than both low (*M*_low_ = 3.35, SD_low_ = 1.42) and high (*M*_high_ = 3.28, SD_high_ = 1.71) intensity, *p* = 0.019 and *p* = 0.012, respectively, while the two did not differ from each other, *p* = 0.792. Thus, gelotophobes did perceive the medium intensity facial expression of the AVATAR as malicious (see medium row on Figure 1). A partial correlation of gelotophobia with maliciousness of the medium intensity expression remained significant even after social phobia (i.e., the SPIN) was partialed out (*r* = 0.18, *p* = 0.04).

Next, we investigated the perception of friendliness. For non-gelotophobes the intensity of the laughter stimuli affected the level of perceived friendliness, *F*(2, 140) = 3.08, *p* = 0.049, η_p_^2^ = 0.042. A trend analysis showed that only the linear trend was significant, *F*(1, 70) = 6.62, *p* = 0.012, η_p_^2^ = 0.086; the friendliness increased with the intensity (*M*_low_ = 4.19, SD_low_ = 1.54, *M*_med_ = 4.56, SD_med_ = 4.56, *M*_high_ = 4.66, SD_high_ = 4.65). No significant effect of intensity was found for the gelotophobes, *F*(2, 58) = 1.77, *p* = 0.180. For them, only the high intensity was perceived as more friendly (*M*_high_ = 4.52, SD_high_ = 1.48), but the difference to the low and medium (*M*_low_ = 4.08, SD_low_ = 1.27, *M*_med_ = 4.08, SD_med_ = 1.37) failed to be significant, *p* = 0.055 and *p* = 0.055 (one-tailed), respectively. Again, a partial correlation between gelotophobia and friendliness of the medium intensity expression was computed (controlling for the SPIN, i.e., social phobia) and it was significant (*r* = 0.21, *p* = 0.03). For the perception of realness, gelotophobia mattered as well. For non-gelotophobes the intensity of the laughter stimuli affected the level of perceived realness, *F*(2, 140) = 8.95, *p* < 0.001, η_p_^2^ = 0.113, and both the medium (*M*_med_ = 4.37, SD_med_ = 1.68, *p* < 0.001) and high (*M*_high_ = 4.23, SD_high_ = 1.63, *p* = 0.003) intensity levels that were perceived higher in realness than the low (*M*_low_ = 3.57, SD_low_ = 1.46) intensity, with the former the two not differing from each other, *p* = 0.487. For the gelotophobes, there was a linear increase of realness with the level of intensity, *F*(1, 29) = 5.80, *p* = 0.023, η_p_^2^ = 0.167, with only the high (*M*_high_ = 4.18, SD_high_ = 1.49) (but not the medium; *M*_med_ = 3.88, SD_med_ = 1.38) intensity level being significantly more real than the low (*M*_low_ = 3.68, SD_low_ = 1.44) intensity level, *p* = 0.023. In other words, once a facial expression was perceived to be “real” (i.e., exceeding the scale midpoint of 4.0), the expressions were significantly exceeding the ones of the lower intensities. This was the case for both medium and high intensity for non-gelotophobes and high intensity only for gelotophobes.

### Analysis of the perception of laughter body movements

Next, we examined the level of realness of the body movements. While the low-intense body movements were considered to be less real (*M*_low_ = 3.03, SD_low_ = 1.29) than the high intensity (*M*_high_ = 5.03, SD_high_ = 1.43), *F*(1, 90) = 6.92, *p* = 0.010, η_p_^2^ = 0.071, the interaction between intensity and gelotophobia failed to be significant, *F*(1, 90) = 1.94, *p* = 0.167. Separate inspection of the five high and five low-intensity examples showed that they varied in intensity and hence the lowest and highest were chosen for further studies. Now, the interaction between gelotophobia and intensity of body movement was significant, *F*(1, 89) = 8.13, *p* = 0.005, η_p_^2^ = 0.084. While the low-intense body movements were considered to be less real by non-gelotophobes (*M*_low_ = 2.64, SD_low_ = 1.65) and (*M*_low_ = 2.73, SD_low_ = 1.82) gelotophobes equally, the non-gelotophobes (*M*_high_ = 5.81, SD_high_ = 1.88) found the high-intense body movement more real than the gelotophobes (*M*_high_ = 4.54, SD_high_ = 2.04). The interaction between intensity and gelotophobia was significant, *F*(1, 89) = 4.81, *p* = 0.031, η_p_^2^ = 0.051. The low-intense body movements were considered to be malicious by both non-gelotophobes (*M*_low_ = 3.15, SD_low_ = 1.69) and gelotophobes (*M*_low_ = 3.15, SD_low_ = 2.11), and while the gelotophobes (*M*_high_ = 3.31, SD_high_ = 1.59) perceived the laughs with the high-intense body movement to be malicious, the non-gelotophobes (*M*_high_ = 2.33, SD_high_ = 1.27), i.e., those that found it real, also stipulated they are not malicious. Finally, the high-intense body display was perceived as friendlier than the laugh with the low-intense body movement, *F*(1, 89) = 8.31, *p* = 0.005, η_p_^2^ = 0.085, and the non-gelotophobes found the laughs more friendly than the gelotophobes did, *F*(1, 89) = 9.69, *p* = 0.002, η_p_^2^ = 0.098. While the non-gelotophobes found the laugh involving the high-intense body movement disproportionately more friendly, the interaction between intensity and level of gelotophobia just failed to be significant, *F*(1, 89) = 3.42, *p* = 0.068. Thus, compared to those without a fear of being laughed at, the gelotophobes found the high-intense body movement less real, more malicious, and less friendly. While, the GELOPH correlated 0.35 (*p* < 0.001) with the perceived maliciousness of the high-intense body movement, controlling for social phobia (i.e., the SPIN) reduced the correlation to a non-significant one (*r* = 0.13, *p* = 0.223).

To explore which body movement features of the feature qualities are linked to perceived as maliciousness in gelotophobes, we next present the two laughter body movement animations that were perceived least and most malicious, respectively, by the gelotophobes. Interestingly, they both came from the high-intensity body movement category (i.e., they even had the same maximal intensity rating in a pretest). This allows for a first comparison that is independent of level of intensity. The means and SD for most malicious were *M* = 3.36, SD = 2.25 and for least malicious *M* = 2.55, SD = 1.47. Table 1 shows the animation that was perceived least malicious and most malicious by the gelotophobes and lists all the body movements that were entailed in these two stimuli. In Supplementary Material, the full video animation can be watched.

**Table 1:** **The body movement, general direction, action type, and action direction for laughter body movement stimuli being perceived as least and most malicious by the gelotophobes**.

Body movement	General direction		Action type	Action direction
	Left to right	Right to left	(AT1–AT13)	(AD1–AD7)
**Least malicious**				
BM 1 weight shifting	No	No	No AT	No AD
BM 2 knees bending	Yes	Yes	Rocking	Back- and forwards
BM 3 abdomen	No	No	Contracting	No AD
BM 4 trunk	Yes	Yes	Rocking	No AD
BM 5 arms	Yes	No	Contracting	Upwards/downwards
BM 6 legs	Yes	Yes	Tilting	No AD
BM 7 chest	No	No	No AT	No AD
BM 8 whole body	Yes	Yes	No AT	Back- and forwards
BM 9 head	Yes	Yes	No AT	No AD
BM 10 shoulders	No	No	No AT	No AD
**Most malicious**				
BM 1 weight shifting	Yes	Yes	No AT	No AD
BM 2 knees bending	No	No	Jerking	Back- and forwards
BM 3 abdomen	No	No	Contracting	Forwards
BM 4 trunk	Yes	No	Tilting	No AD
BM 5 arms	No	No	Jerking	Back- and forwards
BM 6 legs	Yes	Yes	Jerking	Back- and forwards
BM 7 chest	No	No	Contracting	Back- and forwards
BM 8 whole body	No	No	No AT	No AD
BM 9 head	No	No	No AT	Back- and forwards
BM 10 shoulders	No	No	No AT	No AD

*Yes, movement coded; No, no movement coded; AT, action type; AD, action direction following codes are given for the movements and movement directions. Action types: AT1, exhaling; AT2, vibrating; AT3, contracting; AT4, shaking; AT5, tilting; AT6, straightening; AT7, throwing; AT8, jerking; AT9, turning; AT10, rocking; AT11, twitching; AT12, trembling; AT13, convulsing. Body action directions are AD1, backwards; AD2, forwards; AD3, backwards and forwards; AD4, upwards; AD5, downwards; AD6, circular (360°); AD7, curved. If no AD is specified for a BM then only the general direction occurs*.

Table 1 and Supplementary Material show that compared to the least malicious (but intense laughter), there is more jerking than rocking movements in the more malicious laugh and the movement direction is more often forward–backward rather than left–right. Moreover, in the most malicious animation, additionally weight shifts to the left and to the right were observed that were not seen in the least malicious animation. In general, the movements on the most malicious animation appear to be quicker: arms, legs, head are jerking backward and forward, or to left/right, while chest and abdomen are contracted backward or forward. In the least malicious animation, the whole body is contracted while trunk and knees are rocking, and legs are tilting to the left or right.

More precisely, Table 1 and Supplementary Material show that the least malicious body movements involves the bending of the knee, which appear to rock backwards and forwards. The abdomen contracts and moves sideways. The trunk moves in a rocking motion, the arms move left to right, contract upwards then move down again. The legs tilt backwards and forwards, the whole body contracts and head moves from left to right and right to left. In contrast, Table 1 shows that the most malicious laughter involves many weight shifting movements with both from left to right and right to left direction. The knees were seen to bend backwards and forward in a jerking fashion. The abdomen contracted in a vibrating way. The trunk tilting sideways and legs not only moving left to right and right to left but also backwards and forwards. The chest contracted forwards and backwards and the head tilted backwards and forwards. The fact that both are equally intense is also underscored by the fact that for the least malicious movement seven different body movements were coded compared to the eight body movements, which were coded for the most malicious. Interestingly, in the both cases (i.e., the least and the most malicious animation) most of the body parts are involved in laughter expressions including legs, knees, trunk, arm, and head.

### Analysis of auditory stimuli

Owing to the complexity of the variations of the material, we adopted a sequential strategy. First, we computed three repeated measures ANOVAS with the (original and the eight) modification methods as repeated measures (averaged over four stimuli), and the maliciousness, friendliness, and realness ratings as dependent variable for the non-gelotophobia group. If the main effect of different modification methods was significant, *post hoc* tests were used to examine the differences between each of the eight modifications and the original (tests at *p* < 0.05 level). Next, the ANOVA and the *post hoc* tests were repeated in the group of gelotophobes and it was examined whether the same differences exist. Finally, it was examined whether there was a main effect of gelotophobia.

For the non-gelotophobes, the perceived realness of the variations in laughter was significant, *F*(8, 448) = 2.93, *p* = 0.003, η_p_^2^ = 0.046. The *post hoc* tests revealed that adding syllables lead to a higher degree of perceived “realness” of laughter (*p* < 0.001) and reduction of the fundamental frequency by the factor 10 lead to a reduction of the realness of the laughter. For the gelotophobes, the perceived realness of the variations in laughter was significant too, *F*(8, 176) = 6.60, *p* = 0.001, η_p_^2^ = 0.231. The following variations were lowering the realness of the laughter compared to the original adding syllables, reducing the fundamental frequency by the factor 10, and by stretching all phone durations by a factor of 130. Thus, the adding of a syllable has opposite effects for gelotophobes and non-gelotophobes. Finally, the non-gelotophobes gave higher ratings of realness which, however, failed to be significant, *F*(1, 83) = 1.06, *p* = 0.307.

Next, we investigated the perceived maliciousness. The ANOVAs showed that the differences in the auditory laughter stimuli had an impact on perceived maliciousness among both the non-gelotophobes, *F*(8, 664) = 2.93, *p* = 0.003, η_p_^2^ = 0.046 and the gelotophobes, *F*(8, 176) = 2.12, *p* = 0.036, η_p_^2^ = 0.088. *Post hoc* tests revealed that for both groups an increased duration (durations of phones all scaled by factor 130%) was perceived as more malicious compared to the original laughter. Then, for non-gelotophobes, it was the *reduction* (compared to the original) of the variation in the fundamental frequency (F0) that yielded an increase in perceived maliciousness, while for gelotophobes, it was the *amplification* (by 200%) of the variation in the fundamental frequency that was perceived as more malicious. Furthermore, for gelotophobes (but not non-gelotophobes), the linear decrease of the intensity over the laugh episode was perceived as malicious. Finally, the gelotophobes did not generally rate the maliciousness higher than individuals with no fear, *F*(1, 83) = 0.04, *p* = 0.840.

**Figure 2:**
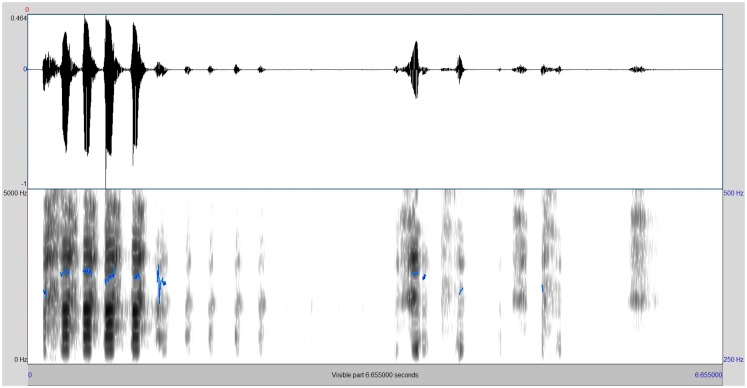
**Spectrogram of the vocal laughter stimulus being perceived as least malicious by the gelotophobes**.

**Figure 3:**
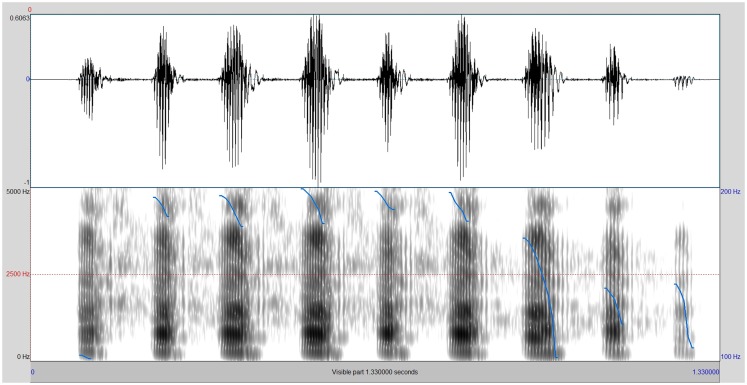
**Spectrogram of the vocal laughter stimulus being perceived as most malicious by the gelotophobes**.

On Figures 2 and 3, the spectrograms of the two stimuli that were perceived as least and most malicious by gelotophobes, respectively, are presented.

The least malicious laugh (as rated by gelotophobes) is shown in Figure 2. Here, a less stereotypical pattern that indicates a natural uninhibited laugh is shown (in this laughter stimulus, the F0 variability was modified). Thus, this finding is in line with former research that indicates the influences of F0 variability on the perception. Figure 3 shows that the stimuli with an increased duration (by stretching all phones by multiplying their duration by the factor 130%) were perceived as most malicious by the gelotophobes. We assume that the stretching of the phones gives the impression of a voluntary regulation/modulation of the laugh and thus makes it sound cognitive.

Finally, the perception of friendliness was investigated. The stimuli differed in the level of perceived friendliness among non-gelotophobes, *F*(8, 448) = 3.54, *p* = 0.001, η_p_^2^ = 0.055, but failed to have a significant overall effect for gelotophobes, *F*(8, 176) = 1.53, *p* = 0.149. *Post hoc* tests revealed that for non-gelotophobes both an increased duration (durations of phones all scaled by factor 130%) and the reduction of the variation in the fundamental frequency (F0) were perceived as less friendly. It should be mentioned that also the shortening of the duration (durations of phones all scaled by factor 70%) and a the linear increase of the intensity over the laugh episode were perceived as less friendly but just failed to be significant (*p* = 0.056). For the gelotophobes, the reduction of the variation in the fundamental frequency (F0) led to a lower perceived friendliness. The amplification of the variation in the fundamental frequency yielded a decrease in friendliness that failed to be significant (*p* = 0.067). Finally, the two groups did not generally differ in their perceived friendliness, *F*(1, 83) = 1.23, *p* = 0.271, although gelotophobes rated all stimuli numerically lower.

### Open answers

The responses to the open question “Which markers in the face/voice/body lead to your perception of friendliness or maliciousness?” were investigated. The first step was to sort each clip of laugh for all modalities by the rating score that had been given by the participant. The laugh was then assigned to group A (more friendly than malicious), group B (more malicious than friendly) or group C (scored equally malicious and friendly).

The analysis of the answers related to the animated facial laughter expression showed that the markers of the friendliness for the laughter in group A were reported as been because of the broadness of the mouth, especially where the teeth was showing, and the raising of the cheeks. In the six animated AVATAR facial expressions, which had been classified as group B, the upper lip curling of the AVATAR and “the eyes” were most often reported as being the markers of maliciousness, these aspects were irrespective of the rater being gelotophobic or not. When participants judged a laugh as been equally friendly and malicious (group C), the reason for this was often the “fakeness” of the virtual agents’ laughter and duration of the laughter facial expression animation.

For the auditory stimuli, the findings of the open answer analysis were in line with the hypotheses: the reasons why laugh sounds were deemed malicious (group B) were given as the slowness or the monotone “ha-ha-ha” sounds. Other examples of the reasons were the “expressionless intonation and lack of variability” and “the tone that sounded controlled.” The group A laughter sounds were described by the participants as being friendly as the laugh sounded “natural” or had a “warm sounding tone.” Additionally, laughs that had a natural trajectory going from high speed to low, which would occur in a usual laughter event, were described as indicating friendliness. As with the facial expression animation, when people judged the laughter sounds equally for malicious and friendly (group C), the reasons given was often due to them being a “fake” or “robotic sound.”

The body movements, which overall were seen as more friendly than malicious, did, however, show that if the clips contained movements, which appeared to be a “pointing” movement of the hand/arm, they were classified as more malicious. When the whole body was moving and the body “leaned backwards” participants rated the laugh as being friendlier.

Second, we looked at the answers of gelotophobes in comparison to individuals with no fear to see whether they nominate any different or additional maliciousness markers beyond the ones where both groups had agreed on. For the facial expression, while the non-gelotophobes rated the lip curls as markers of maliciousness and eyes as markers of friendliness, the gelotophobes nominated the eyes as markers of maliciousness and the lips, mouth, and teeth as markers of friendliness. Thus, indicating a reverse pattern of the features that generate the perception of maliciousness and friendliness.

Furthermore, for the auditory laughter the non-gelotophobes often gave the high pitch of the laughter as being the indicator of maliciousness. The gelotophobes commented that the “fakeness,” so the artificial sound (particularly slow, particularly long), made it appear malicious. Friendliness was often based on the brevity or shortness of the laughter sound for the non-gelotophobes and its “fastness” was indicated as making the sound friendly for the gelotophobes.

For the body movements, non-gelotophobes said that when the body appeared “stiff” it appeared as malicious. The gelotophobes saw the “stillness” of the body as being malicious. When it was rated friendly, the shoulder and head movements were nominated as markers for the non-gelotophobes whereas the gelotophobes did not nominate any observable features of what determines friendliness in laughter body movements.

## Discussion

Gelotophobia is a specific disposition that biases the perception of joyful stimuli (expressed not only by laughter but also beyond). As laughter is an integral part of interaction it is important to create virtual agents that can account for such biases, by producing laughter that is perceived as non-malicious also to those individuals with gelotophobia. Furthermore, there are features of laughter that are perceived as malicious by both, gelotophobes and non-gelotophobes. If the desired encoding of the virtual laughter is malicious, then these features should be significantly reduced.

The current study identified features of facial, vocal, and body laughter stimuli that were perceived as malicious in general, and for gelotophobes specifically. In general, our hypothesis that “cognitive” laughs would be perceived as more malicious was confirmed for the face and the voice. While for non-gelotophobes, maliciousness did not vary with intensity of the facial expression, the gelotophobes were sensitive to the intensity of the display. The analysis of the laughter facial displays showed that gelotophobes mostly perceived the mid intensity stimuli as malicious. The mid level intensity laughs shows that amusement is present but probably down regulated and not at maximum. This might indicate a cognitive element of attempting to dampen (or hiding imperfectly) amusement. For the perception of realness, gelotophobia mattered as well. The low-intensity laugh was considered to be the least real, and the highest intensity as most real. For gelotophobes, there was a linear increase and for non-gelotophobes the middle intensity was as real as the high intensity. Once a facial expression was perceived to exceed the scale midpoint of 4.0 (i.e., is perceived as “real”), the expressions were significantly exceeding the ones of the lower intensities. Overall, it has to be mentioned that the gelotophobes did not differ from the individuals with no fear in the friendliness and maliciousness ratings for the facial laughter stimuli. However, it was clear from the open answers that the triggers for those ratings did differ, in fact, they were the opposite of each other. Namely, that the shape and appearance of the lips curling induced feelings that the expression was malicious for non-gelotophobes and that the movement round the eyes, elicited the face to appear as friendly. The converse was true for the gelotophobes. The lips were what made the appearance of the virtual agent friendlier and the eyes made the appearance seem malicious. This is interesting, as it is speculated that gelotophobes are “laugh blind” in as much as they do not have a feeling for what the sender of the expression is trying to relate. As the contraction of the orbicularis oculi muscle is what differentiates a cognitive from a real expression of enjoyable emotion (Ekman et al., 1990) or williness to cooperation (Schug et al., 2010), the fact that movement around the eye is indeed deemed malicious would be problematic and lead to misinterpretation of facially expressed communication and may be one reason gelotophobes find it more difficult to form or maintain long-term adult relationships than non-gelotophobes do (Platt et al., 2010; Platt and Forabosco, 2012). Most importantly, the effect found for maliciousness cannot be explained by social anxiety, i.e., is specific to gelotophobia. A different but plausible pattern emerged for friendliness, which was a linear function increased for non-gelotophobes. For the gelotophobes, only the high intensity was perceived as more friendly.

For the body laughter stimuli, the intensity of body movement did play a role. While the low-intense body movements were considered to be less real, there was an interaction between gelotophobia and intensity of body movement (i.e., the lowest and highest in intensity) with the non-gelotophobes finding the high-intense body movement more real than the gelotophobes. It was also the non-gelotophobes finding the high-intense body movement less malicious (and friendlier) than the gelotophobes did and how both groups perceived the low-intensity laughs, i.e., those that found it real, also stipulated they are not malicious. Thus, compared to those without a fear of being laughed at, the gelotophobes found the high-intense body movement less real, more malicious, and less friendly. Furthermore, to modify AVATAR laughter displays to be suitable for gelotophobic individuals, we looked at the single stimuli in details and studied the laughter body movement animations that had received the lowest and highest maliciousness ratings, respectively (gelotophobic participants only). This qualitative, descriptive analysis showed that the laughter stimulus that displayed an uninhibited, strong laugh was perceived as least malicious. The stimulus that went along with less and slower body movements and higher retrained body movements was perceived most malicious. This is in line with our hypothesis that perceived cognitive modulation increases the perceived maliciousness (laughter as an explicit communication attempt, not felt emotion). Additionally, the weight shift and the more frequent back and forth movements might be perceived as more threatening than the sideward directions found for the low-malicious laughter. In the open answers, the non-gelotophobes stated that for them the quality that gave the body movement its maliciousness was stiffness. This was not picked up by the gelotophobes. Yet, going stiff and “feeling paralyzed” is an item on the GELOPH <15 > (Ruch and Proyer, 2008a). It has also been reported by extreme gelotophobes in case-study responses. When asked the question What do you experience when you feel being laughed at, among other things, they often reported body stiffness (Platt, under review). It could be for the gelotophobes, this body movement relates more to being fearful rather than been malicious.

For the perception of auditory laughter features, stretching the duration of the syllables compared to the original laughter was perceived as more malicious irrespective of gelotophobia level. While for the non-gelotophobes maliciousness was because of the *reduction* of the variation in the fundamental frequency, maliciousness was because of the *amplification* for gelotophobes. Similarly, the gelotophobes saw maliciousness in the linear decrease of the intensity over the laugh episode. Variations in the acoustics made a difference for friendliness among non-gelotophobes only. A reduction of friendliness could be obtained by an increase (and decrease) in duration, a reduction of the variation in the fundamental frequency, and a linear increase of the intensity over the laugh episode. For the gelotophobes, only the reduction of the variation in the fundamental frequency (F0) tended to lower perceived friendliness. It seems that several deviations from the sound of spontaneous laughter gives the impression, or indicates the “evil mind” behind the laughter interferes and that presumably disparaging thoughts (about the gelotophobe), which in the gelotophobe’s view add volitional elements and render an emotional laughter to one that carries the “you are ridiculous” message. The parameters reported above need to be considered when designing a laughing AVATAR in the future. Right now, we do not know how much of these deviations from the normal laugh is tolerable and there was also no test of the interaction between the different parameters. Furthermore, it should also be remembered that already the normal laughter did yield an average level of maliciousness (*M*_non-g_ = 3.06, SD_non-g_ = 1.27, *M*_g_ = 3.12, SD_g_ = 1.16).

To summarize, a gelotophobia-friendly laugh should consist of a high intensity, uninhibited facial expression, containing the Duchenne markers [see Ekman et al. (1990) and Ruch and Ekman (2001)], a voiced vocalization, which is fast, non-repetitive, variable, and of short duration. It should not contain any features that indicate a down-regulation in the voice or body, or indicate cognitive.

### Limitations

This is the first study to investigate the fine-grained laughter features in different modalities to identify fear triggers of AVATAR laughter in gelotophobes. Still, due to the huge amount of stimuli, we were limited to only 6 (respective 10) stimuli for the face and body, with the stimuli only being distinguished by the dimension of intensity. For the face, only one AVATAR was utilized, which was only of female gender and thus any effects of the AVATAR appearance or gender on the perception could not be investigated. Concerning the analysis of laughter body movements, this was to our knowledge the first psychological study that investigated the laughter body movement perception of gelotophobes. Future studies should attempt more fine-grained analysis to identify which exact features and movement trajectories are linked to perceived maliciousness. Automatic feature analysis with many samples of laughter stimuli should be correlated to the subjective ratings. Here, we could only attempt a descriptive analysis of the feature qualities. Still, our results deliver first evidence of which features may be modified when generating gelotophobia-friendly AVATAR laughter body movements. Furthermore, this study considered the three modalities independently. Future studies should also focus on the importance of audiovisual integration in the perception of laughter friendliness/maliciousness.

One has to consider that people with a fear of being laughed at are rare. For example, in Switzerland only 5% of the population measured is gelotophobic. So to get a sample of 20 you need to sample over 400 participants. Additionally, gelotophobes are difficult to find for studies relating to laughter, as this is the trigger of fear, panic, and feelings of shame. Getting gelotophobes, especially those at the more pathological levels, to fully commit to such a study, even with the guarantee of absolute anonymity, is not easily achieved. Building trust by hosting face-to-face, rather than through online testing, could encourage more participation but this would limit wider participation. Presenting the modalities in the lab may encourage more of the rare extreme gelotophobes cases to undertake the task, as they could be reassured and encouraged.

## Conflict of Interest Statement

The authors declare that the research was conducted in the absence of any commercial or financial relationships that could be construed as a potential conflict of interest.

## Supplementary Material

The Supplementary Material for this article can be found online at http://www.frontiersin.org/Journal/10.3389/fnhum.2014.00928/abstract

## Footnotes

^1^*Pitch*: note that in speech processing literature, and given the nature of the signals, pitch is assimilated with fundamental frequency or the vibration with the lowest frequency

^2^*Intensity*: to modify the intensity pattern of the laugh, the amplitude of each audio sample was multiplied by a weighting factor. This factor was either decreasing or increasing linearly over the laugh episode, with a maximum value of 1 (at the beginning or end of the laugh, respectively) and a minimal value of I (at the end or beginning of the laugh, respectively). Laughs have been synthesized for the factor 10 (increase and decrease). The unmodified original laugh corresponds to I = 1. *Rhythm*. Rhythm was modified by multiplying with the same factor *R* the duration of each phone in the base phonetic transcription, then synthesizing the obtained phonetic transcription. Factors smaller than 1 shorten the laugh (faster rhythm). Laughs were synthesized with values of *R* equaling 70 or 130% (reference laugh corresponding to *R* = 1). *F0*. To investigate the impact of fundamental frequency (F0) patterns, the F0 curves from the base synthesis laughs have been altered. The average F0 of each base synthesis laugh was computed, and the deviations from the average value were multiplied by a factor *F*. Fundamental frequency variations are amplified if *F* > 1 and attenuated if *F* < 1 (*F* = 0 corresponding to a constant fundamental frequency over the laugh). For each base synthesis laugh, F0 was altered by the factor 10% (reduction) or 200% (amplification). *Syllables*. To vary the number of syllables, we looked for the longest series of “fricative-vowels” syllables (FV series) in the base laugh transcription. To obtain laughs with smaller numbers of syllables, we deleted syllables in the series in a uniform way: if only 1 syllable has to be deleted, we select the middle syllable from the base FV series; if 2 syllables have to be dropped, we take them at 1/3 and 2/3 of the base FV series; if 3 syllables are removed, we take them around 1/4, 1/2, and 2/3 of the base FV series. To obtain laughs with higher numbers of syllables, we added syllables in the FV series in a uniform way. The duration of the inserted syllable is the average between the durations of the preceding and following syllables, while the vowel of the inserted syllable is copied from the preceding syllable. For each base synthesis laugh, five syllables were either added or removed.

^3^The MMLI corpus consists of approximately 500 laugh episodes from 16 participants. It contains both induced and interactive laughs from human triads. The motion capture (mocap) data consist of 3D body position information, multiple audio and video channels, as well as respiration data. The intensity of MMLI laugh episodes was annotated by 2 coders who used a 3-step scale (low, medium high).

^4^The two instruction conditions did not impact on the ratings (no main effects or interactions) and were thus neglected in further analyses.
